# Cecropin-Loaded Zeolitic Imidazolate Framework Nanoparticles with High Biocompatibility and Cervical Cancer Cell Toxicity

**DOI:** 10.3390/molecules27144364

**Published:** 2022-07-07

**Authors:** Jingwen Jiang, Yanzhu Pan, Jinyao Li, Lijie Xia

**Affiliations:** Xinjiang Key Laboratory of Biological Resources and Genetic Engineering, College of Life Science and Technology, Xinjiang University, Urumqi 830017, China; jjw@stu.xju.edu.cn (J.J.); 990911pyz@stu.xju.edu.cn (Y.P.)

**Keywords:** cecropins, zeolitic imidazolate framework, nanoparticles, antitumor

## Abstract

Cecropins (CECs) are insect venom-derived amphiphilic peptides with numerous pharmacological effects, including anti-inflammatory, antibacterial, antiviral, and anti-tumor activities. Cecropins induce tumor cell death by disrupting phospholipid membrane integrity. However, non-specific cytotoxicity and in vivo rapid degradation limit clinical application. Nanotechnologies provide novel strategies for tumor eradication, including nanocarriers that can precisely target drugs to tumor tissue. We report the fabrication of CEC-encapsulated zeolitic imidazolate framework 8 (ZIF-8) nanoparticles (CEC@ZIF-8 NPs) via the preparation of CEC@ZIF-8 NPs in pure water by one-pot stirring. This method yielded morphologically uniform NPs with 20 wt% drug loading capacity and 9% loading efficiency. The NP formulation protected CECs from proteasome degradation, enhanced peptide bioavailability, promoted HeLa tumor cell uptake, and increased antitumor efficacy compared to free CECs. In conclusion, this ZIF-8 encapsulation strategy may enhance the clinical applicability of CECs and other antitumor peptides.

## 1. Introduction

While total cancer-related mortality is decreasing, a substantial survival gap has emerged between industrialized and developing countries [1]. Cervical cancer rates, for example, are higher in developing countries, accounting for about 85% of global cases. Cervical cancer also accounts for a relatively greater proportion of cancer-related deaths in developing countries. The most important factor causing cervical cancer is human papillomavirus (HPV) infection, which can be divided into high pathogenic and low pathogenic genotypes. Cervical cancer is mainly treated by surgery, drug therapy, radiation therapy, and immunotherapy [2]. The prognosis of metastatic, recurrent, and other advanced cervical cancer is still very poor [3]. In fact, the five-year survival rate of women with stage III cervical cancer fell to 30–40%, while that of women with stage IV cervical cancer was only 15% or less [4]. Therefore, it is urgent to develop new strategies to improve the clinical efficacy of cervical cancer treatment.

Most anticancer drugs have limited selectivity for cancer cells over surrounding normal cells, especially normal cells with high mitosis rates [5]. Therefore, it is imperative to develop methods for targeted delivery of anticancer drugs to tumor sites. Among the most promising delivery vehicles are nanoparticles (NPs). For instance, metal–organic frameworks (MOFs) are recently developed porous organic-inorganic hybrid nanomaterials with particularly advantageous for drug delivery due to their simple preparation methods, high drug loading capacity, and functional biodegradability [6,7]. Toxicity studies on various MOFs have shown that the ligands constituting MOFs are basically nontoxic, and different metal species will produce different toxicity [8]. Zeolites imidazole ester skeletons (zifs) are a unique kind of MOFs, which have been widely studied in recent years. Several MOFs including UiO (University of Oslo) [9], MI (material from Institut Lavoisier), and zeolitic imidazolate frameworks (ZIFs) have been used to load drugs. ZIF-8 is a typical ZIF, which is composed of zinc ions and 2-methylimidazole (MIM) [10]. Of the ZIF family, ZIF-8 is considered the most promising for drug delivery due to better drug loading capacity and higher thermal stability, chemical stability, and biocompatibility than many other MOFs [11,12,13]. Most importantly, ZIF-8 can retain embedded components under neutral (physiological) pH but release these components in weakly acidic environments such as tumors, thereby achieving controlled and targeted release of anticancer drugs [14]. Therefore, ZIF-8 NPs are among the most widely studied MOFs in medical research.

Antimicrobial peptides (AMPs), also known as host defense peptides, are important components of many immune systems. These AMPs usually range from 10 to 50 amino acids in length [15] and most have a net positive charge, usually +2 to +9, due to the presence of Lys and Arg residues [16,17]. Various AMPs demonstrate broad-spectrum toxicity against bacteria [18,19], fungi [20,21], viruses [22,23], and cancers [24,25], as well as pro-inflammatory and anti-inflammatory activities through effects on innate and adaptive immunity [26], and effects on autophagy, apoptosis, and hemostasis [27,28,29,30]. Unlike traditional chemotherapy drugs, insect AMPs have specific cytotoxicity to tumor cells, but have no obvious toxic side effects on normal human cells, and have a cumulative effect on the treatment of cancer [31]. It has recently been discovered that modified AMPs will overcome the limitations of stability, activity, and toxicity associated with natural AMPs [32].

Cecropins (CECs) are antibacterial peptides of the sericin-B family first isolated from the 3rd instar larvae of the *Bombyx mori* in Aksu, Xinjiang. The mature peptide is a kind of Cationic polypeptide and contains 37 amino acids and includes an amphiphilic α-helix region. Cecropins have a wide antibacterial spectrum [33] and also inhibit the growth of various tumor cell lines, including human gastric cancer BGC823 cells in vitro and in vivo [34,35]. However, free antimicrobial peptides cannot be administered systemically due to poor bioavailability. Although various attempts have been made to encapsulate antimicrobial peptides in inorganic-based carriers such as quantum dots (QDs) and NPs [36] or in organic-based carriers such as liposomes [37], the synthesis methods are relatively complex and time-consuming. Therefore, it is highly desirable to develop a simple and efficient methodology for the incorporation of antimicrobial peptides such as CEC into simple inorganic nanocarriers.

In this study, ZIF-8 NPs were used to load CECs and prepared with water as the medium, which were named CEC@ZIF-8 NPs. The characteristics of CEC@ZIF-8 NPs were investigated, including structure, loading capacity and efficiency, protection of the peptide, and bioavailability. We demonstrate that encapsulation of CECs into ZIF-8 NPs increases both intracellular accumulation and cytotoxicity in cervical cancer cells.

## 2. Results and Discussion

### 2.1. Evaluation of Drug Incorporation and Characterization of CEC@ZIF-8 NP Morphology

**Figure 1:**
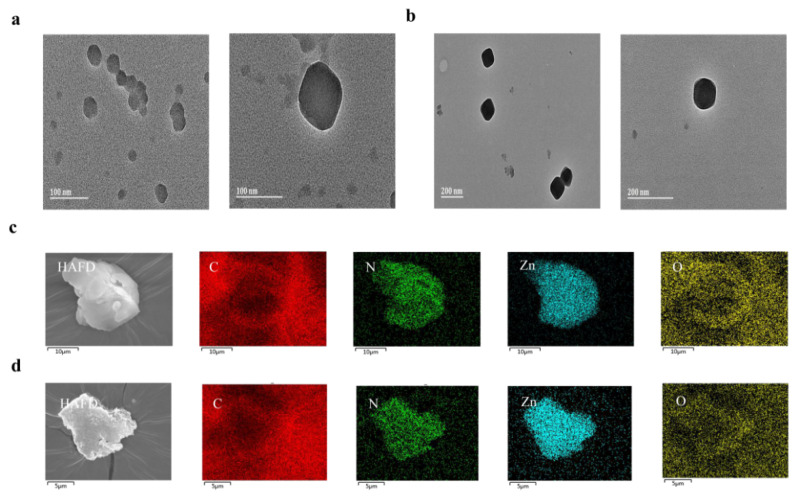
Size distributions and shapes of ZIF-8 NPs (**a**) and CEC@ZIF-8 NPs (**b**) as revealed by transmission electron microscopy. EDX mapping of ZIF-8/CEC@ZIF-8 NPs (**c**,**d**): Zn, C, O, and N element.

**Scheme 1:**
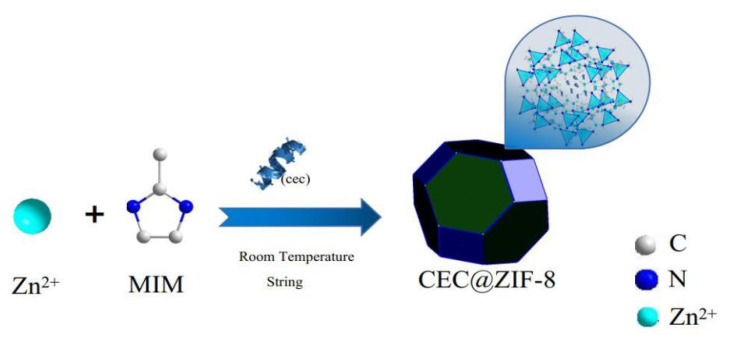
Construction of a nano drug delivery system based on ZIF-8.

In the present work, the natural anticancer peptide CEC was encapsulated in ZIF-8 NPs via simple, rapid, and universal reaction steps under mild conditions to preserve bioactivity (Scheme 1). The CEC@ZIF-8 NPs were prepared by crystallization of ZIF-8 NP precursors (zinc nitrate and 2-methylimidazole) with CEC under stirring. Figure 1 presents TEM images of typical ZIF-8 NPs (Figure 1a) and CEC@ZIF-8 NPs (Figure 1b). The average size of the pure ZIF-8 NPs was about 100 nm and encapsulation of CEC increased the average particle size to about 150 nm, possibly due to enhanced growth kinetics mediated by CEC-seeded clusters [38]. These elemental mapping images also reveal that the main elements (C, N, O, Zn) were randomly distributed in the CEC@ZIF-8 NPs (Figure 1c,d).

**Figure 2:**
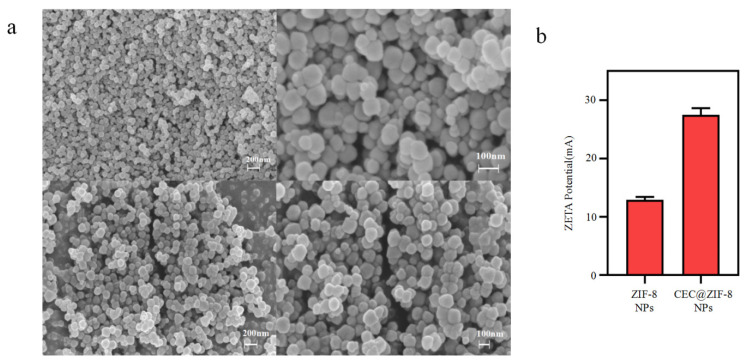
Surface morphologies and zeta potentials of ZIF-8 NPs and CEC@ZIF-8 NPs. (**a**) Scanning electron micrographs of ZIF-8 NPs (upper) and CEC@ZIF-8 NPs (lower), and their partial enlarged drawing (right). The pictures were selected from at least three independent experiments with similar results. (**b**) Zeta potentials of ZIF-8 NPs and CEC@ZIF-8 NPs. Data are presented as the mean ± SD from three independent tests.

Morphologically, both ZIF-8 NPs and CEC@ZIF-8 NPs were nearly rhombic dodecahedrals ranging in diameter from 100–150 nm as revealed by SEM (Figure 2a). The surfaces of both NPs were relatively smooth, indicating that CEC molecules were largely encapsulated within the NP crystalline structure. Furthermore, the zeta potential was changed from +12.9 to +27.5 mV by encapsulated CEC (Figure 2b), indicating that the cationic peptides were successfully integrated into the framework structure of ZIF-8.

**Figure 3:**
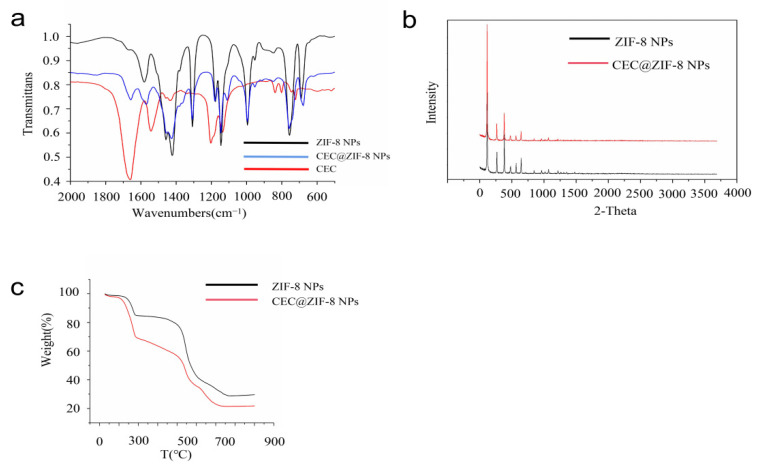
Elemental and molecular structure analyses demonstrating incorporation of intact CEC into the crystalline structure of ZIF-8 NPs. (**a**) FTIR spectra of ZIF-8 NPs, CEC@ZIF-8 NPs, and free CEC. (**b**) XRD spectra of ZIF-8 NPs and CEC@ZIF-8 NPs. (**c**) TGA curves for ZIF-8 NPs and CEC@ZIF-8 NPs.

Fourier transform infrared (FTIR) spectroscopy was performed to characterize the chemical reactions involved in CEC incorporation (Figure 3). The FTIR spectrum of CEC@ZIF-8 NPs was similar to that of ZIF-8 NPs (Figure 3a) except for a new peak at l651 cm^−1^, which could be attributed to carbonyl stretching as suggested by previous FTIR analyses of ZIF-8 NP drug loading [39,40]. The FTIR spectrum of bare ZIF-8 nanoparticles exhibited peaks at ~1573 cm^−1^ attributable to C=N stretching vibration, at ~1140 cm^−1^ attributable to C–N stretching vibration, and at ~420 cm^−1^ attributable to Zn–N stretching vibration [41,42]. The CEC spectrum included the same peak at ~l651 cm^−1^ as CEC@ZIF-8 NPs and both materials showed the C-O absorption peak at 1100–1250 cm^−1^, COOH absorption peak at 750–800 cm^−1^, and NH^2+^ peak at 800–850 cm^−1^ common to peptides [43], demonstrating that intact CEC was incorporated into the ZIF-8 NP crystals.

To further illustrate the homogeneity of ZIF materials in NPs, we conducted a powder X-ray diffraction analysis (Figure 3b). The ZIF-8 diffraction pattern exhibited a number of peaks attributable to a cubic structure with an I-43 m space group [44], and the diffraction pattern of CEC@ZIF-8 was identical, with no additional diffraction peaks due to low CEC crystallization. The diffraction peaks of both ZIF-8 and CEC@ZIF-8 were also sharp and intense, indicating highly crystalline structures. The similarity between ZIF-8 and CEC@ZIF-8 suggests that CEC loading is homogenous and has no impact on its crystal structure.

**Figure 4:**
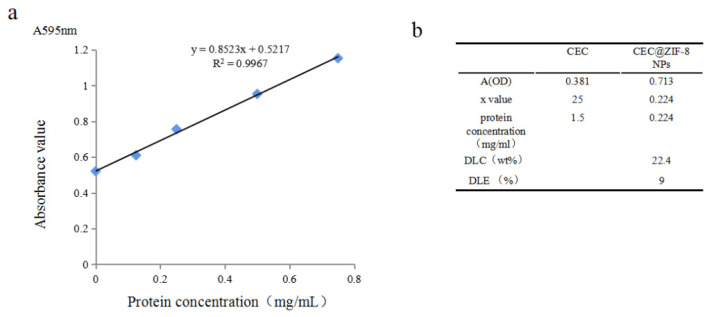
Loading capacity and efficiency of ZIF-8 NPs for CEC. (**a**) Standard curve for the Bradford protein assay. (**b**) DLC and DLE of CEC@ZIF-8 NPs.

Thermogravimetric analysis (TGA) of ZIF-8 NPs in air atmosphere (Figure 3c) revealed a long plateau in the range from room temperature to 450 °C, indicating high thermal stability. In contrast, the TGA curve of CEC@ZIF-8 NPs exhibited a gradual weight loss of 20% over this same range, implying that about 20% of the NP weight is CEC loaded into the framework. Protein quantitation using a Bradford assay also yielded a loading efficiency of 20 wt% as well as an encapsulation efficiency of 9% (Figure 4), consistent with TGA analysis.

**Figure 5:**
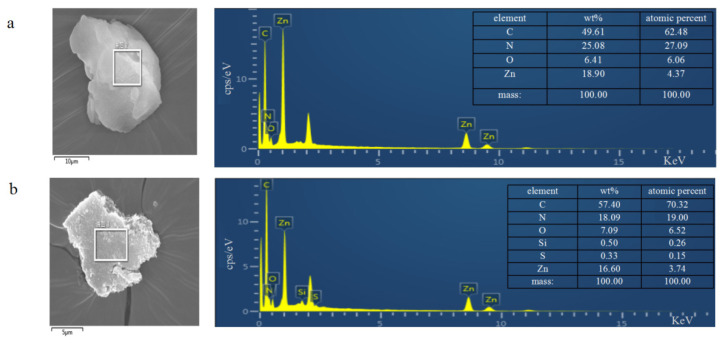
(**a**) Electron microscopy images of ZIF-8 NPs and EDX images of ZIF-8 NPs. (**b**) Electron microscopy images of CEC@ZIF-8 NPs and EDX analysis of CEC@ZIF-8 NPs.

In addition, energy-dispersive X-ray (EDX) analysis confirmed the presence of the expected elements in both ZIF-8 and CEC@ZIF-8 NPs (Figure 5). Carbon (C), nitrogen (N), and zinc (Zn) from 2-methimidazole (organic compounds) and six zinc nitrates (metal part) were found in both ZIF-8 and CEC@ZIF-8 NPs. The uniform dispersions of C, N, O, and Zn elements within the NP structure were observed. Moreover, EDX analysis showed that the C/N ratio was increased from 2.3 to 3.6 by the inclusion of CEC in the synthesis reaction, further confirming the incorporation of CEC into the crystal structure of ZIF-8 NPs.

### 2.2. Uptake of Nanoparticles into Tumor Cells

**Figure 6:**
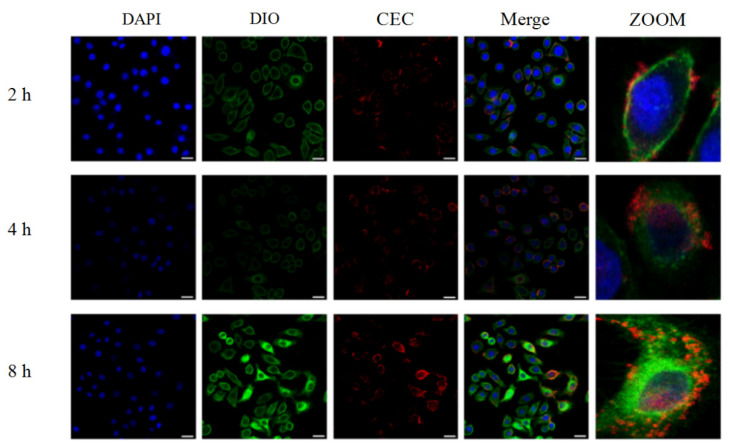
Uptake of Nile red-conjugated CEC@ZIF-8 NPs into HeLa cells (scale bar: 25 µm). Cytoplasmic and membrane accumulation of NR-CEC@ZIF-8 NPs (red fluorescence) increased with time during incubation for 2, 4, and 8 h. Membranes were stained with DIO (green) and nuclei with DAPI (blue).

To examine the cellular uptake potential, Nile red (NR) conjugates of free CEC and CEC@ZIF-8 NPs were prepared and used to treat HeLa cells, then intracellular accumulation was monitored by fluorescence confocal microscopy using a 543 nm laser [45]. At the same time, HeLa cell nuclei were stained with DAPI (blue fluorescence), and cell membranes were stained with DIO (green fluorescence) to examine potential membrane disruption by CEC. Cells were treated with NR-CEC@ZIF-8 NPs at a CEC content of 50 µg/mL for 2, 4, and 8 h (Figure 6). After 2 h of treatment, weak red fluorescence signals were observed in the cytoplasm and cell membrane but mainly co-localized with the green fluorescent membrane, indicating that some NPs had inserted into the membrane, and with longer incubation, the co-localized red fluorescence signals gradually increased. After 4 h, membrane vesicles started to appear and the initially well-delineated membranes gradually became indistinct, suggesting structural degradation. After 8 h, many cells exhibited membrane vesicles and broken membranes that were the characteristics of cell-penetrating peptides. Cationic polypeptides, generally rich in basic amino acid residues, interact with negatively charged phospholipids (on the cell membrane) through electrostatic attraction to form holes and break the cell membrane [44,45,46]. The charge load on the surface of tumor cells is much stronger than that of normal cells, so antimicrobial peptides generally have a strong hydrophobic effect with phospholipid on the tumor cell membrane, showing a good anticancer effect [47].

### 2.3. Effect of CEC@ZIF-8 Nanoparticles on Tumor Cell Viability

**Figure 7:**
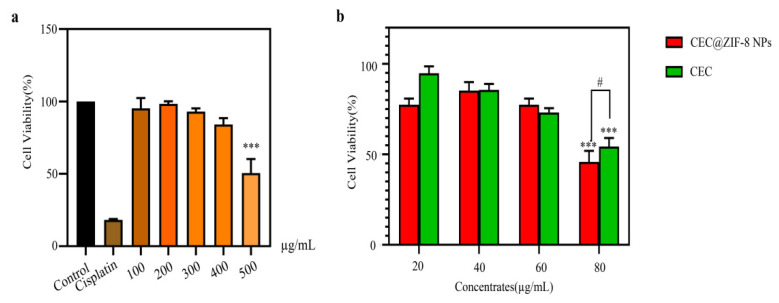
The cytotoxicity of ZIF-8 and CEC@ZIF-8 NPs against HeLa cells. (**a**) Minimal cytotoxicity of ZIF-8 NPs. Unloaded ZIF-8 NPs had little effect on HeLa cell number, suggesting good biocompatibility and safety as a drug nanocarrier. *** *p* < 0.001 vs. control group. (**b**) CEC@ZIF-8 incorporation of CEC into ZIF-8 NPs increases cancer cell toxicity. The cytotoxicity of CEC@ZIF-8 NPs against HeLa cells was significantly greater than that of free CEC at equivalent concentrations. Since CEC@ZIF-8 NPs have a 20% loading capacity, the maximum dosage of ZIF-8 NP corresponding to 500 μg/mL equates to CEC@ZIF-8 100 μg/mL. Data are presented as the mean ± SD from three independent tests. *** *p* < 0.001 compared to the 20 μg/mL treatment group. # *p* < 0.05 compared to the same concentration of CEC treatment group.

To examine the potential antitumor efficacy of CEC@ZIF-8 NPs, we conducted MTT assays. HeLa cells were incubated with the indicated concentrations of ZIF-8 NPs, free CEC, CEC@ZIF-8 NPs, vehicle (negative control), or cisplatin (positive control) for 24 h. Survival rates exceeded 80% at all doses of ZIF-8 NPs compared to negative controls (Figure 7a), indicating that ZIF-8 NPs possess negligible cytotoxicity within this concentration range. This result is consistent with previous studies that demonstrated the biocompatibility and safety of ZIF-8 NPs as drug nanocarriers for cancer therapy [48,49]. Incorporation of CEC into ZIF-8 NPs increased cytotoxicity compared to equivalent concentrations of free CEC with 80 μg/mL (*p* < 0.05) (Figure 7b), suggesting that the nanocarrier greatly enhances CEC effective endocytosis and accumulation into the cytoplasm [43].

## 3. Materials and Methods

### 3.1. Materials

Cecropin was synthesized by Shanghai Ketai Biotechnology (97%). Dimethylimidazole (97%) was purchased from Shanghai Yuanye Biological Technology. Zinc nitrate hexahydrate (97%) and ultra-pure water were also used for synthesis.

### 3.2. Synthesis of ZIF-8 Nanoparticles

First, 1.0 g of zinc nitrate hexahydrate dissolved in 10 mL deionized water was added to 19.4 g of 2-methylimidazole dissolved in 90 mL deionized water under agitation at room temperature to form a mixture with a zinc ion:2-methylimidazole:water molar ratio of 1:70:1653. After 1 min, the mixture turned milky white, indicating that ZIF-8 nanoparticles had begun to form. The solids were then removed, washed 5 times in deionized water to remove unreacted reagents, freeze-dried, and stored at −20 °C.

### 3.3. Synthesis of CEC@ZIF-8 Nanoparticles

A 1.0 g sample of zinc nitrate hexahydrate dissolved in 10 mL deionized water and 20 g of 2-methylimidazole dissolved in 90 mL deionized water with 200 mg CEC were added together under stirring at room temperature to form a mixture with a zinc ion:2-methylimidazole:water molar ratio of 1:70:1653. The mixed solution turned milky white after stirring for 1 min, indicating that ZIF-8 crystals had begun to form. To ensure complete crystallization and improve the CEC loading, the reaction was continued for 5 min. Solids were then separated by centrifugation (14,000 rpm, 10 min), washed three times in deionized water, freeze-dried, and stored at −20 °C.

### 3.4. Characterization of Unloaded and CEC-Loaded NPs

Both unloaded and CEC-loaded NPs were characterized by scanning electron microscopy (SEM), transmission electron microscopy (TEM), X-ray diffraction (XRD), Fourier transform infrared absorption spectroscopy (FTIR), zeta potential analysis, and thermogravimetric analysis (TGA). The morphology and size of ZIF-8 NPs and CEC@ZIF-8 NPs were characterized by 100 kV field emission TEM. Briefly, NPs were suspended in methanol/aqueous solution and dispersed by ultrasound. A 10 mL sample of the suspension was dripped onto a copper wire covered with carbon film, air-dried at room temperature, and photographed. For SEM analysis, the suspensions were spread onto double carbon strips containing copper root, coated with gold using an SCD050 BAL-TEC sputter coater under a vacuum, and imaged using a Shimadzu ss-550 SEM equipped with a tungsten filament. In addition, FTIR was conducted from 500–2000 cm^−1^ on NPs suspended in potassium bromide using a Bruker Vertex 70 Fourier transform infrared spectrophotometer. Finally, TGA was conducted using a NetzchSta 449 C system. The test range was from room temperature to ~800 °C at a heating rate of 10 °C/min in an air atmosphere.

### 3.5. Determination of CEC@ZIF-8 NPs Loading and Encapsulation Rates

Drug loading and encapsulation rates were calculated according to the following equations.

Drug loading content efficiency (DLC) = NP-loaded CEC mass/CEC@ZIF-8 NP mass × 100%

Drug loading encapsulation efficiency (DLE) = NP-loaded CEC mass/total CEC mass in reaction × 100%

### 3.6. Confocal Laser Scanning Microscopy

The internalization of NPs into cultured tumor cells was examined by confocal microscopy using Nile red (NR) as a fluorescent tracer. For the preparation of NR-labeled NPs, 1 mg Nile red dissolved in 2.5 mL ethanol solution was added to 1.0 g zinc nitrate hexahydrate dissolved in 10 mL deionized water under stirring at room temperature, followed by ethanol volatilization and further mixing with a solution of 19.4 g 2-methylimidazole and 200 mg CEC in 90 mL ultrapure water. Again, the solid was removed, washed three times in deionized water, freeze-dried, and stored at −20 °C for later use. Tumor-derived HeLa cells were treated with free CEC, ZIF-8 NPs, and CEC@ZIF-8 NPs as indicated for 2, 4, and 8 h. After washing with PBS, cells were fixed with 4% ice-cold paraformaldehyde at 4 °C for 10 min, stained with DIO (Beyotime, Haimen, China) at 37 °C for 15 min and DAPI (Beyotime, China) at room temperature for 5 min, and observed by confocal microscopy (Nikon A1R HD25 Confocal Microscope, Eclipse Ti-E Series, Tokyo, Japan). The image processing software used for analysis was Nikon Eclipse.

### 3.7. Cell Lines and Cell Culture

The human cervical cancer cell line HeLa was obtained from the Xinjiang Key Laboratory of Biological Resources and Genetic Engineering, Xinjiang University (Urumqi, Xinjiang, China) and cultured in Roswell Park Memorial Institute (RPMI) 1640 medium (Gibco, Thermo Fisher Scientific, Waltham, MA, USA) supplemented with 10% fetal bovine serum (MRC, Changzhou, China) and 1% of l-glutamine (100 mM), 100 U/mL penicillin, and 100 µg/mL streptomycin (MRC, Changzhou, China) at 37 °C humidified air with 5% CO_2_.

### 3.8. Cell Viability Assay

The effects of CEC@ZIF-8 NPs and free CEC on HeLa cell viability were examined using the 3-(4,5-dimethyl-2-thiazolyl)-2,5-diphenyl-2-H-tetrazolium bromide (MTT) cell counting assay (Sigma, MO, USA). Cells at the logarithmic growth phase were seeded in 96-well culture plates at 5 × 10^3^ cells/well with 100 μL of cell medium. The medium was replaced the following day with fresh medium containing CEC@ZIF-8 NPs (20, 40, 60, and 80 μg/mL), equal concentrations of free CEC diluted from the stock solution, or vehicle (negative control). The clinical antitumor agent cisplatin (35 g/mL) was used as a positive control. After 24 h, cell culture plates were centrifuged at 1200 rpm for 5 min, the supernatant discarded, and 100 μL of MTT solution (0.5 mg/mL in PBS) added to each well. Cells were incubated for an additional 3 h at 37 °C. Cell culture plates were then centrifuged at 1200 rpm for 7 min, and the supernatant was discarded. The formazan crystals formed from MTT by viable cells were dissolved in 200 μL DMSO and the optical density values at 490 nm (OD490) were measured on a 96-well microplate reader (Bio-Rad Laboratories, Hercules, CA, USA). All treatments were conducted in triplicate. Cell viability was calculated using the following formula:

Cell viability (%) = (Absorbance in test wells/Absorbance in negative control wells) × 100%

## 4. Conclusions

We have developed a simple and efficient method for the preparation of water-dispersible cecropin-loaded ZIF-8 nanoparticles. These CEC@ZIF-8 NPs had an acceptable CEC loading capacity (20 wt%) and encapsulation efficiency (9%). Characterization showed that CEC@ZIF-8 was regular (dodecahedral), uniform in size, effectively loaded into the vector, and possessed characteristics necessary for clinical drug delivery vectors. In vitro studies demonstrated that encapsulation of CEC in ZIF-8 NPs greatly enhanced intracellular accumulation and toxicity against a cervical cancer cell line. This work highlights the potential of MOFs as simple, stable, and highly efficient drug delivery vehicles for cancer treatment.

## Data Availability

Not applicable.
